# Sustainability of assistive technology use for students with severe and multiple disabilities: a mixed methods study of teachers’ attitudes and experiences

**DOI:** 10.3389/fpsyg.2026.1930074

**Published:** 2026-08-27

**Authors:** Hülya Torun Yeterge

**Affiliations:** Department of Special Education, Faculty of Education, Adıyaman University, Adıyaman, Türkiye

**Keywords:** severe and multiple disabilities, assistive technology, special education teachers, sustainability, teacher attitudes, explanatory sequential mixed methods

## Abstract

**Introduction:**

Assistive technologies play a vital role in supporting the communication, learning, independence, and participation of students with severe and multiple disabilities. However, their effectiveness does not necessarily ensure their long-term and sustainable use in educational settings. While previous research has primarily focused on the effectiveness of assistive technologies, limited attention has been paid to teachers’ attitudes and experiences regarding their sustainable implementation. This study aimed to examine the sustainable use of assistive technologies in the education of students with severe and multiple disabilities from the perspectives of teachers’ attitudes and experiences.

**Methods:**

An explanatory sequential mixed methods design was employed. Quantitative data were collected from 132 special education teachers working with students with severe and multiple disabilities. Subsequently, semi-structured interviews were conducted with 20 teachers selected from the higher and lower ends of the attitude-score distribution. Quantitative data were analyzed using descriptive and inferential statistics, including an independent-samples t-test, while qualitative data were analyzed through thematic analysis.

**Results:**

Teachers demonstrated generally positive attitudes toward assistive technologies (M = 73.63/90). However, qualitative findings revealed that positive attitudes alone were insufficient to ensure sustainable use. Institutional support, technical infrastructure, professional development opportunities, and school-level implementation processes emerged as the key factors influencing sustainability.

**Discussion:**

The findings suggest that the sustainable use of assistive technologies cannot be explained solely by technology acceptance. Rather, teachers’ psychological dispositions should be considered alongside organizational and contextual factors. The study contributes to the literature by providing a more comprehensive understanding of sustainable assistive technology use and offers practical implications for teacher education, school leadership, and educational policy.

## 1 Introduction

Assistive technology (AT) is widely recognized as one of the fundamental tools for supporting the daily living, communication, learning, and social participation of individuals with disabilities (Mukhtarkyzy et al., 2025). In educational settings, assistive technologies facilitate students’ access to instructional activities, promote their independence, and provide learning opportunities tailored to their individual needs (Abdelwahab et al., 2025). Recent advances in digital technologies have considerably expanded the scope of assistive technologies, leading to the widespread use of a variety of solutions in educational practice, including augmentative and alternative communication (AAC) systems, accessible digital materials, environmental control systems, and microswitch applications (Beirat et al., 2025). Consequently, assistive technologies are now regarded not only as supportive tools but also as essential components of inclusive and accessible education (Kooli and Chakraoui, 2025; Sousa et al., 2025).

The importance of assistive technology becomes even more evident in the education of students with severe and multiple disabilities (Lancioni et al., 2025). Severe and multiple disabilities refer to a complex disability condition characterized by the co-occurrence of two or more disabilities across cognitive, sensory, and physical domains, requiring intensive and ongoing support (Şafak, 2019; Westling et al., 2020). A variety of assistive technologies are used in the education of students with severe and multiple disabilities, including augmentative and alternative communication (AAC) systems, eye-gaze technologies, microswitch systems, and tablet-based applications. These technologies constitute essential educational tools that support students’ communication, participation, and independence (Lancioni et al., 2013). The significant challenges these students experience in communicating, interacting with their environment, moving independently, and participating in learning activities necessitate individualized instructional practices and the systematic use of assistive technologies. Through the use of assistive technologies, students’ active participation in educational activities can be enhanced, independent living skills can be promoted, and access to learning opportunities can be improved.

A substantial body of research has demonstrated that assistive technologies provide significant educational benefits for students with severe and multiple disabilities (Çay and Bozak, 2025). These technologies have been reported to enhance students’ communication skills, increase their interaction with the environment, support their independence, strengthen academic participation, and improve their overall quality of life (Çay et al., 2020). However, the majority of existing studies have focused primarily on the effectiveness of assistive technologies in improving specific skills, while giving limited attention to how these technologies are sustained over the long term in educational settings and the factors influencing the continuity of their use. Demonstrating the educational effectiveness of assistive technologies does not necessarily indicate that they are routinely and sustainably used in educational environments. Previous research has shown that although many assistive technologies are initially adopted, their frequency of use declines over time, and some technologies are abandoned before they can be fully integrated into educational practice (Federici and Scherer, 2018; Phillips and Zhao, 1993). Therefore, the educational effectiveness of assistive technologies should not be interpreted as evidence that they will be used consistently and sustainably in educational settings over the long term.

Sustainability in education refers not only to the adoption of effective practices but also to their becoming an integral part of routine instructional practices over time, maintaining their applicability across different educational contexts, and receiving institutional support (Moore et al., 2017). In this context, the sustainable use of assistive technologies is shaped by the interaction of numerous individual and organizational factors, including technical infrastructure, maintenance and updating processes, institutional support, opportunities for professional development, and user experiences (Tondeur et al., 2017). Therefore, the successful integration of assistive technologies into educational systems depends not only on their accessibility but also on their long-term integration into educational practice. From this perspective, the key question shifts from *“Does this technology work?”* to *“Why is this technology sustained in educational settings, or why is it abandoned?”* The long-term use of assistive technologies is closely associated with teachers’ adoption of these technologies, their integration into instructional processes, their incorporation into routine teaching practices, and their continued use despite the challenges encountered. Accordingly, sustainability should be viewed as a dynamic process shaped not only by the characteristics of the technology itself but also by users’ behaviors and the organizational context in which the technology is implemented.

The sustainable use of assistive technologies in educational settings and their integration into routine instructional practices for students with severe and multiple disabilities largely depend on the special education teachers who directly use these technologies (Ertmer and Ottenbreit-Leftwich, 2010). The same assistive technology may be used with varying frequency and in different ways by different teachers. This indicates that sustainable use cannot be explained solely by technological infrastructure; teachers’ attitudes, beliefs, and experiences with technology also play a critical role. As the primary implementers of assistive technologies, teachers integrate these technologies into instructional processes, maintain their routine use, and adapt them to meet students’ individual needs. Therefore, understanding teachers’ psychological dispositions toward assistive technologies is essential for explaining the sustainable use of these technologies in educational settings.

Several theoretical approaches have been developed to explain the sustainable use of assistive technologies in educational settings. One of the most widely adopted theoretical frameworks for explaining teachers’ technology use is the Technology Acceptance Model (TAM), which posits that individuals’ intention to use a technology is largely determined by their perceived usefulness and perceived ease of use (Davis, 1989; Venkatesh and Bala, 2008). Studies in educational settings have likewise demonstrated that teachers’ attitudes toward technology are among the most influential factors affecting technology integration (Scherer et al., 2019; Teo, 2011). However, technology adoption alone does not guarantee its long-term use. The sustainability of educational practices depends not only on individuals’ adoption of new practices but also on their ability to integrate these practices into daily instructional processes, incorporate them into routine teaching practices over time, and continue using them by adapting to changing conditions (Moore et al., 2017). The Normalization Process Theory (NPT) provides a useful framework for explaining this process by suggesting that new practices become sustainable when individuals make sense of the practice (*coherence*), engage with it (*cognitive participation*), integrate it into their routine practices (*collective action*), and continuously evaluate and refine its implementation (*reflexive monitoring*) (May and Finch, 2009; May et al., 2009). Taken together, these two theoretical perspectives suggest that the sustainable use of assistive technologies is influenced by both teachers’ psychological dispositions and the organizational context in which implementation takes place.

Although positive teacher attitudes toward assistive technologies may facilitate their integration into classroom practice, how these positive attitudes translate into long-term and sustainable use remains insufficiently understood. In other words, teachers’ adoption of assistive technologies and their continued use over time do not represent the same process (Ertmer and Ottenbreit-Leftwich, 2010; Luo et al., 2025; Scherer et al., 2019). Empirical evidence explaining how these two processes unfold, particularly in the context of students with severe and multiple disabilities, remains limited. Existing studies in special education have primarily focused either on describing teachers’ attitudes toward assistive technologies or on examining the effects of these technologies on students with severe and multiple disabilities (Çay and Bozak, 2025). However, little is known about how teachers’ attitudes shape the sustainable use of assistive technologies, the psychological and contextual mechanisms underlying this process, or how sustainability is experienced in the education of students with severe and multiple disabilities. Addressing these issues requires mixed methods research that integrates quantitative and qualitative evidence to provide a more comprehensive understanding of this complex phenomenon (Creswell and Plano Clark, 2018). Determining teachers’ attitudes toward assistive technologies alone is insufficient to explain why sustainable use occurs. A deeper understanding of how these attitudes are translated into classroom practices and influenced by individual and organizational factors requires qualitative findings to complement and explain quantitative results.

Against this background, the present study aimed to examine the sustainability of assistive technology use in the education of students with severe and multiple disabilities through an explanatory sequential mixed methods approach, focusing on teachers’ attitudes and experiences. Specifically, the study sought to identify special education teachers’ attitudes toward assistive technologies and to explore how teachers with different attitude levels perceive the factors that facilitate or hinder the sustainable use of these technologies. By integrating quantitative and qualitative findings, the study aimed to provide a comprehensive understanding of the psychological and contextual factors that support or constrain the long-term use of assistive technologies in educational settings.

Accordingly, the study addressed the following research questions:

1. What are the levels of special education teachers’ attitudes toward assistive technologies for students with severe and multiple disabilities?
2. How do teachers with high levels of positive attitudes perceive the sustainable use of assistive technologies?
3. How do teachers with low levels of positive attitudes perceive the sustainable use of assistive technologies?
4. In what ways do the views of teachers with high and low attitude levels differ regarding the sustainable use of assistive technologies?

## 2 Method

### 2.1 Research design

**Figure 1:**
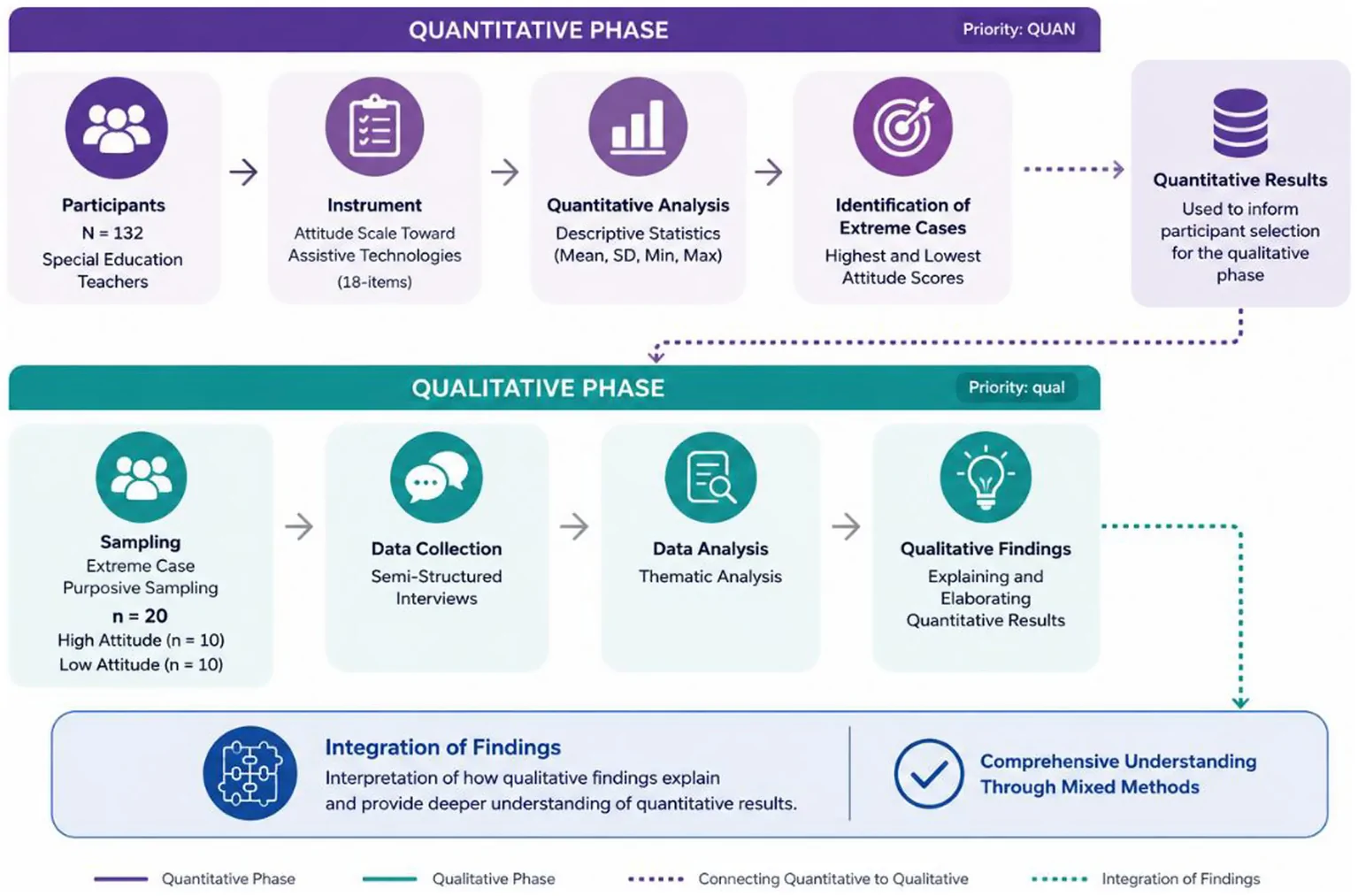
Explanatory sequential mixed methods design of the study.

This study was conducted using an explanatory sequential mixed methods design to examine the sustainability of assistive technologies used in the education of students with severe and multiple disabilities based on teachers’ attitudes and experiences. The explanatory sequential mixed methods design is a mixed methods approach that involves collecting and analyzing quantitative data in the first phase of the research process, followed by the collection of qualitative data in the second phase to provide a more in-depth explanation of the quantitative findings (Creswell and Plano Clark, 2018). This design enables researchers to identify general trends related to a particular phenomenon through quantitative data and subsequently explain the underlying reasons for these trends and their contextual reflections through qualitative data. In this study, the attitudes of special education teachers toward assistive technologies were first determined. Subsequently, through interviews conducted with teachers who had high and low attitude scores toward assistive technologies, the factors affecting the sustainable use of assistive technologies were examined in detail. Thus, the study aimed to provide a holistic understanding of teachers’ attitudes and their experiences regarding the sustainable use of assistive technologies (see Figure 1).

### 2.2 Participants

A total of 132 special education teachers working with students with severe and multiple disabilities in different provinces of Türkiye participated in the quantitative phase of the study. Participants were selected using criterion sampling, one of the purposive sampling methods. Criterion sampling aims to select individuals who meet predetermined criteria directly related to the research problem (Patton, 2015). In the present study, the primary criterion was that teachers were actively working with students with severe and multiple disabilities. This approach was adopted to obtain experiences regarding the sustainable use of assistive technologies from participants who had direct experience and expertise in the subject.

In the qualitative phase of the study, participant selection was informed by the quantitative findings, consistent with the explanatory sequential mixed methods design. The total scores obtained by the 132 teachers on the Attitude Scale Toward Assistive Technologies were ranked, and teachers from the higher and lower ends of the score distribution were contacted sequentially and invited to participate. When a teacher declined participation, the next teacher in the corresponding score ranking was contacted. Interviews continued until data saturation was reached, that is, until additional interviews no longer contributed substantively new codes or themes. Data saturation was achieved with a total of 20 teachers, comprising 10 teachers from the higher end and 10 teachers from the lower end of the attitude-score distribution. This approach enabled a comparative examination of the experiences of teachers with contrasting attitude scores regarding the sustainable use of assistive technologies (Table 1).

**Table 1:** Demographic characteristics of teachers participating in the quantitative phase (*N* = 132).

Variable	Category	*n*	%
Gender	Female	82	62.1
	Male	50	37.9
Professional experience	1–5 years	28	21.2
	6–10 years	41	31.1
	11–15 years	34	25.8
	16 years or more	29	22.0
Educational level	Bachelor’s degree	103	78.0
	Master’s degree	29	22.0
Institution type	Special Education Practice School	132	100
Received assistive technology training	Yes	57	43.2
	No	75	56.8

Table 2 presents the demographic characteristics of the teachers who participated in the qualitative phase of the study. The qualitative sample was formed based on the total scores obtained from the Attitude Scale Toward Assistive Technologies. First, the teachers were ranked according to their total attitude scores. Teachers with the highest and lowest scores were then contacted sequentially and invited to participate in the qualitative phase. If a teacher declined to participate, the next teacher in the ranked list was contacted. Interviews continued until data saturation was reached, which occurred with 20 teachers, including 10 teachers from the higher end and 10 teachers from the lower end of the attitude-score distribution. The high-attitude group had a mean score of 89.40 (SD = 0.52; range = 89–90), whereas the low-attitude group had a mean score of 52.70 (SD = 0.68; range = 52–54). This sampling strategy enabled a comparison of teachers’ experiences regarding the sustainable use of assistive technologies across different attitude levels.

**Table 2:** Characteristics of teachers participating in the qualitative phase (*N* = 20).

Participant code	Gender	Professional experience	Institution type	Attitude group
K1	Female	8 years	SES	High
K2	Female	12 years	SES	High
K3	Male	6 years	SES	High
K4	Female	15 years	SES	High
K5	Male	9 years	SES	High
K6	Female	13 years	SES	High
K7	Female	7 years	SES	High
K8	Male	18 years	SES	High
K9	Female	10 years	SES	High
K10	Male	14 years	SES	High
K11	Female	4 years	SES	Low
K12	Male	5 years	SES	Low
K13	Female	3 years	SES	Low
K14	Female	6 years	SES	Low
K15	Male	8 years	SES	Low
K16	Female	5 years	SES	Low
K17	Male	11 years	SES	Low
K18	Female	7 years	SES	Low
K19	Male	4 years	SES	Low
K20	Female	9 years	SES	Low

SES = Special Education Practice School.

### 2.3 Data collection instruments

The quantitative data of the study were collected using the Attitude Scale Toward Assistive Technologies, developed by Aslan and Kan (2017). The scale was developed to assess teachers’ attitudes toward assistive technologies and consists of 18 items and four subscales in a five-point Likert-type format. Items are rated on a five-point Likert scale, yielding a total score ranging from 18 to 90, with higher scores indicating more favorable attitudes toward assistive technologies. The subscales include the behavioral component, affective component, negative affect component, and cognitive component. The scale development study was conducted with 638 participants, and the four-factor structure was confirmed through exploratory and confirmatory factor analyses. The overall Cronbach’s alpha internal consistency coefficient of the scale was 0.88, while the alpha coefficients for the subscales ranged from 0.71 to 0.83. These findings indicate that the scale is a valid and reliable instrument for measuring teachers’ attitudes toward assistive technologies (Aslan and Kan, 2017). For the present sample (*N* = 132), the internal consistency of the Attitude Scale Toward Assistive Technologies was recalculated. The scale demonstrated excellent internal consistency (Cronbach’s *α* = 0.93).

The qualitative data were collected using a semi-structured interview form developed by the researcher. While developing the interview questions, consideration was given to the sustainable use of assistive technologies, teachers’ experiences, institutional support mechanisms, challenges encountered during implementation, and recommendations for enhancing sustainability. To establish the content validity of the interview form, opinions were obtained from three academics with expertise in special education, educational technology, and qualitative research. The interview questions were revised in accordance with their recommendations.

### 2.4 Data collection procedure

In the quantitative phase of the study, data were collected through an online survey. Participants were informed about the purpose of the study, informed consent was obtained on a voluntary basis, and subsequently the Personal Information Form and the Attitude Scale Toward Assistive Technologies were administered. Following the analysis of the quantitative data, participants for the qualitative phase were selected based on their attitude scores. The interviews were conducted either online or face-to-face, depending on the participants’ preferences, and each interview lasted approximately 30–45 min. The total interview duration was approximately 790 min. With the participants’ permission, all interviews were audio-recorded and subsequently transcribed verbatim.

### 2.5 Data analysis

Quantitative data were analyzed using descriptive statistics, including mean, standard deviation, minimum, and maximum scores. In addition, an independent-samples t-test was conducted to examine whether teachers’ total attitude scores differed according to their AT-specific training status. Prior to the analysis, the assumptions of the independent-samples t-test, including the distribution of scores, potential outliers, and homogeneity of variances, were examined.

The qualitative data were analyzed using the thematic analysis approach proposed by Braun and Clarke (2006). First, the interview transcripts were read repeatedly to achieve familiarity with the data. Subsequently, meaningful data segments were coded. Similar codes were then grouped to generate themes, which were reviewed and refined in accordance with the research questions. Finally, the themes were defined and interpreted, and the findings were supported with direct quotations from the participants.

To enhance the consistency of the qualitative analysis, a code–recode strategy was employed. The qualitative data were coded by the researcher at two different points in time, and the coding decisions were subsequently compared to examine consistency. Any discrepancies identified between the two coding rounds were revisited and resolved through re-examination of the relevant data segments.

### 2.6 Integration of quantitative and qualitative findings

The integration process, which constitutes an important stage in mixed methods research, was carried out during both participant selection and the interpretation of the findings (Creswell and Plano Clark, 2018). Participants in the qualitative phase were selected based on the attitude scores obtained in the quantitative phase, thereby establishing a direct connection between the two datasets. Furthermore, in the discussion section, the quantitative findings and qualitative themes were interpreted together to provide a holistic understanding of the factors influencing the sustainable use of assistive technologies.

### 2.7 Validity, reliability, and trustworthiness

In accordance with the methodological rigor required in mixed methods research, several strategies were employed to enhance validity, reliability, and trustworthiness throughout both the quantitative and qualitative phases of the study (Creswell and Plano Clark, 2018). In the quantitative phase, the Attitude Scale Toward Assistive Technologies, whose validity and reliability had previously been established (Aslan and Kan, 2017), was employed. In addition, missing and erroneous data as well as outliers were examined during the data entry process to ensure the accuracy of the dataset before analysis. The same data collection instrument was administered to all participants to ensure standardization of the measurement process.

In the qualitative phase, the criteria of credibility, transferability, dependability, and confirmability proposed by Lincoln and Guba (1985) were adopted. The semi-structured interview form was developed based on the opinions of three academics with expertise in special education, assistive technologies, and qualitative research. Following a pilot implementation with two teachers, the interview questions were revised accordingly. During the interviews, probing questions were used to enrich the data, all interviews were transcribed verbatim, and member checking was conducted by asking participants to review the summaries of their interviews.

To enhance confirmability, researcher notes were maintained throughout the research process, analytical decisions were documented, and the findings were supported by direct quotations from the participants. To strengthen transferability, detailed descriptions of the study group, the research context, and the data collection and analysis procedures were provided. To ensure the methodological validity of the mixed methods design, participants in the qualitative phase were selected based on the attitude scores obtained during the quantitative phase. Quantitative and qualitative data were integrated and interpreted using a joint display approach. This process contributed to a more comprehensive and trustworthy interpretation of the findings regarding the sustainable use of assistive technologies.

Since the study was conducted by a single researcher, reflexivity was maintained throughout the qualitative analysis by critically reviewing coding decisions and revisiting the data during the code–recode process. In this context, the interview transcripts were read multiple times at different stages of the analysis, the coding process was reviewed repeatedly at regular intervals, and the initial coding was compared with subsequent coding to evaluate consistency. Throughout the coding and theme development process, the analysis remained closely aligned with the research questions. Analytical decisions were systematically documented through researcher notes, and the findings were supported by direct participant quotations to ensure that the interpretations were grounded in the data.

### 2.8 Ethical considerations

This study was approved by the Scientific Research and Publication Ethics Committee of the Faculty of Social and Human Sciences at Adıyaman University (decision no. 32, Date: 17 February 2026). Prior to data collection, informed consent was obtained from all participating teachers on a voluntary basis. Participants’ identities were kept confidential, all data were anonymized before analysis, and the data were used solely for scientific purposes.

## 3 Results

### 3.1 Quantitative findings

In the first phase of the study, the attitudes of special education teachers working with students with severe and multiple disabilities toward assistive technologies were examined. Descriptive statistics for the scores obtained from the Attitude Scale Toward Assistive Technologies are presented in Table 3.

**Table 3:** Descriptive statistics for teachers’ attitude scores toward assistive technologies (*N* = 132).

Variable	*N*	Mean	SD	Min	Max
Total attitude score	132	73.63	12.70	52	90

As shown in Table 3, the participating teachers obtained a mean total attitude score of 73.63 (SD = 12.70), with scores ranging from 52 to 90. The mean score suggests a generally favorable tendency toward assistive technologies. Based on the quantitative findings, teachers with the highest and lowest total attitude scores were identified. Among these teachers, those who volunteered to participate were included in the qualitative phase of the study. This procedure enabled the quantitative findings to be further explored through qualitative data, consistent with the explanatory sequential mixed methods design.

An independent-samples t-test was conducted to examine whether teachers’ attitudes toward assistive technologies differed according to AT-specific training. Teachers who had received AT-specific training (*n* = 57, M = 75.18, SD = 13.19) had slightly higher attitude scores than those who had not received such training (*n* = 75, M = 72.45, SD = 12.27); however, the difference was not statistically significant, t(130) = 1.22, *p* = 0.224, Cohen’s d = 0.21.

### 3.2 Qualitative findings

The thematic analysis of the qualitative data revealed that teachers’ experiences and perspectives regarding the sustainable use of assistive technologies were organized under four main themes. These themes were identified as (1) the educational value of assistive technologies, (2) factors supporting sustainable use, (3) factors limiting sustainability, and (4) recommendations for sustainable use. During the development of the themes, the views of teachers with high and low attitude scores were compared, enabling the similarities and differences regarding the sustainable use of assistive technologies to be identified from a holistic perspective. The findings were supported by direct quotations from the participants under each theme.

Table 4 presents the themes and subthemes identified through the qualitative analysis, together with the frequency of participants referring to each code. The findings indicate that teachers’ perspectives on the sustainable use of assistive technologies were organized under four main themes. The most frequently reported codes were related to the educational value of assistive technologies and the factors supporting or limiting their sustainable use. These themes were developed by comparatively examining the views of teachers with high and low attitude levels.

**Table 4:** Themes and subthemes derived from the qualitative findings.

Theme	Subtheme	*n*
Educational value of assistive technologies	Supporting communication	17
	Increasing classroom participation	15
	Supporting independence	14
Factors supporting sustainability	In-service training	16
	Teacher experience	13
	Institutional support	12
Factors limiting sustainability	Lack of technical support	18
	Device malfunctions	15
	Time constraints	12
	Lack of training	17
Recommendations for sustainable use	In-service training	18
	Technical support	16
	Policy development	14

*n* indicates the number of participants who referred to each subtheme. Because a participant could be associated with more than one subtheme, the total frequency exceeds the total number of participants.

#### 3.2.1 Theme 1: educational value of assistive technologies

The majority of teachers in the high-attitude group (*n* = 9, 90%) stated that assistive technologies are indispensable educational tools that support the communication, independence, classroom participation, and motivation of students with severe and multiple disabilities. A considerable proportion of teachers in the low-attitude group (*n* = 8, 80%) also acknowledged the educational benefits of assistive technologies; however, they reported experiencing various difficulties during their implementation. Participants particularly emphasized the positive effects of assistive technologies on communication skills, independence, classroom participation, and motivation. While teachers in the high-attitude group described assistive technologies as “an indispensable part of education,” teachers in the low-attitude group also recognized their benefits but indicated that they encountered various challenges during implementation. One teacher described this situation as follows: *“For some of my students, communication is almost impossible without assistive technologies. In particular, visual and auditory supports significantly increase students’ participation in classroom activities”* (P4).

#### 3.2.2 Theme 2: factors supporting sustainable use

The majority of teachers in the high-attitude group (*n* = 9, 90%) reported that the most important factors supporting the sustainable use of assistive technologies were teacher experience, practice-oriented training, and institutional support. Approximately half of the teachers in the low-attitude group (*n* = 5) stated that they would be able to use assistive technologies more effectively if adequate training and support were provided. Teachers in the high-attitude group particularly emphasized that practical training and experience in using technology enhanced sustainability. According to these teachers, assistive technologies gradually become a natural part of instructional practices over time. One participant expressed this view as follows: *“The more I use them, the easier they become. At first, they seemed difficult, but now they have become an integral part of my lesson plans”* (P8). Teachers in the low-attitude group also stated that they would use assistive technologies more frequently if sufficient support were available.

#### 3.2.3 Theme 3: factors limiting sustainability

Participants reported numerous barriers that hindered the sustainable use of assistive technologies. In particular, the lack of technical support, device malfunctions, financial constraints, limited time, and insufficient in-service training were frequently mentioned by both groups. Teachers in both the high-attitude group (*n* = 10, 100%) and the low-attitude group (*n* = 10, 100%) referred to various barriers limiting the sustainable use of assistive technologies. However, the majority of teachers in the high-attitude group (*n* = 8, 80%) perceived these challenges as manageable, whereas most teachers in the low-attitude group (*n* = 9, 90%) considered the same challenges to be major obstacles that substantially limited the continued use of assistive technologies. One teacher described this challenge as follows: *“When a tablet breaks down, it waits for months to be repaired. Eventually, teachers give up using it”* (P17). Another participant stated: *“We have assistive technology, but there is no one to teach us how to use it. Without training, the devices end up sitting in cupboards”* (P13).

#### 3.2.4 Theme 4: recommendations for sustainable use

The majority of participants (*n* = 18, 90%) emphasized that the sustainable use of assistive technologies in educational settings requires system-level improvements rather than relying solely on individual efforts. An examination of teachers’ views revealed that their recommendations were concentrated in three main areas. First, participants emphasized that practice-oriented in-service training should be provided regularly and continuously to support the effective and sustainable use of assistive technologies. Second, they recommended the availability of technical support personnel in schools, the acceleration of maintenance and repair processes, and the establishment of support mechanisms capable of providing timely solutions to the technical problems encountered by teachers. Finally, participants stressed that access to assistive technologies should not depend solely on teachers’ individual initiatives but should instead be strengthened through sustainable practices supported by school administrations, relevant institutions, and national educational policies. These findings indicate that the sustainable use of assistive technologies depends not only on teachers’ positive attitudes but also on the structural and institutional support provided by the education system. One participant summarized this perspective as follows: *“We want to use assistive technology; however, for its use to be sustainable, teachers’ willingness alone is not enough. Regular training, technical support, and the school’s commitment to this process are essential. Otherwise, after a while, the devices stop being used”* (P8).

### 3.3 Integration of quantitative and qualitative findings

Consistent with the mixed methods design of the study, the quantitative and qualitative findings were interpreted together. The quantitative findings indicated that teachers generally held positive attitudes toward assistive technologies. However, the qualitative findings revealed that these positive attitudes alone did not guarantee the sustainable use of assistive technologies. While the quantitative findings indicated a generally favorable tendency toward assistive technologies, the qualitative findings showed that sustainable use was influenced not only by teachers’ attitudes but also by contextual factors, including technical support, in-service training, institutional resources, and support from school administration.

Table 5 presents the joint display integrating the quantitative and qualitative findings of the study. The table comparatively presents the quantitative findings, the qualitative findings that explain them, and the integrated interpretations derived from combining both datasets. This integration illustrates the relationship between the quantitative and qualitative findings regarding the sustainable use of assistive technologies.

**Table 5:** Integration of quantitative and qualitative findings.

Quantitative findings	Qualitative findings	Integrated interpretation
The mean attitude score indicated a generally favorable tendency toward assistive technologies (M = 73.63/90).	Teachers reported that assistive technologies made significant contributions to communication, participation, independence, and learning processes.	Teachers’ high attitude scores are explained by their positive experiences regarding the educational benefits of assistive technologies for students.
Teachers generally held positive attitudes toward assistive technologies.	Teachers in both the high- and low-attitude groups stated that assistive technologies were necessary and beneficial.	There is a general consensus among teachers regarding the educational value of assistive technologies.
Teachers expressed willingness to use assistive technologies.	Teachers, particularly those in the high-attitude group, described assistive technologies as a natural part of their instructional practices.	Positive attitudes support teachers’ willingness to integrate assistive technologies into instructional practice.
Although teachers’ attitude scores were generally high, some participants obtained relatively lower scores.	Lack of technical support, device malfunctions, time constraints, and insufficient in-service training were identified as the main barriers to sustainable use.	Although positive attitudes are necessary for sustainable use, technical and institutional barriers may limit the continuity of technology use.
Teachers demonstrated positive attitudes toward assistive technologies.	Participants emphasized the importance of school administration support, technical support, and continuous professional development opportunities.	The sustainability of assistive technologies depends not only on individual attitudes but also on institutional support mechanisms.
High- and low-attitude groups differed quantitatively.	Teachers in the high-attitude group perceived the challenges encountered as manageable, whereas teachers in the low-attitude group considered the same challenges to be significant barriers.	Teachers’ attitudes influence their perceptions of the sustainable use of assistive technologies and their approaches to coping with implementation challenges.
The mean attitude score indicated a generally favorable tendency toward assistive technologies.	Even teachers with high attitude scores reported technical and institutional problems.	Although positive attitudes are necessary for sustainable use, they are not sufficient on their own.

## 4 Discussion

This study examined the attitudes of special education teachers working with students with severe and multiple disabilities toward assistive technologies and their experiences regarding the sustainable use of assistive technologies in education using a mixed methods approach. The quantitative findings indicated a generally favorable tendency toward assistive technologies among teachers. However, the qualitative findings revealed that although positive attitudes constitute an important factor supporting the sustainable use of assistive technologies, they are not sufficient on their own. Rather, sustainability is shaped by the interaction of multiple technical, institutional, and pedagogical factors.

The quantitative analysis further showed that teachers who had received AT-specific training had somewhat higher attitude scores than those who had not received such training; however, this difference was not statistically significant. This finding suggests that participation in AT-specific training alone may not be sufficient to account for differences in teachers’ attitudes toward assistive technologies. Rather, the qualitative findings indicate that the sustainability of AT use may depend on broader factors, including the practical relevance and continuity of training, access to technical support, institutional conditions, and opportunities to integrate AT into everyday educational practices. Thus, the findings underscore the importance of considering training as part of a broader support system rather than as an isolated factor in promoting sustainable AT use.

The first finding of the study indicates that teachers generally hold positive attitudes toward assistive technologies. The high mean scores obtained from the attitude scale suggest that teachers are aware of the importance of assistive technologies in the education of students with severe and multiple disabilities and have developed positive perceptions of these technologies. This finding is consistent with previous studies examining special education teachers’ attitudes toward assistive technologies (Aslan and Kan, 2017; Demirok et al., 2019; Memet and Şentürk, 2021). In particular, teachers working with students with severe and multiple disabilities may have developed positive attitudes toward assistive technologies through their direct experiences of the important role these technologies play in improving students’ communication, participation, and independent living skills.

This finding can also be interpreted within the framework of the Technology Acceptance Model (TAM). According to Davis (1989), individuals’ intention to use a technology is largely shaped by their perceptions of its usefulness and ease of use. During the qualitative interviews, teachers emphasized that assistive technologies increased students’ classroom participation, facilitated communication, and supported their learning processes, indicating a high level of perceived usefulness of assistive technologies. This may be considered an important factor explaining the positive attitude scores obtained in the quantitative phase.

However, the qualitative findings of the present study revealed that although positive attitudes are necessary for the sustainable use of assistive technologies, they are not sufficient on their own. This interpretation is consistent with previous research suggesting that teachers’ technology-related beliefs and attitudes interact with broader pedagogical and contextual factors in shaping technology use (Petko, 2012). Teachers with both high and low attitude levels acknowledged the educational value of assistive technologies; however, they also reported the existence of various barriers to their sustainable use. In particular, the lack of technical support, problems related to the maintenance and repair of devices, limited financial resources, and insufficient in-service training were frequently identified by the teachers. This finding suggests that the long-term use of assistive technologies in educational settings cannot be explained solely by individual willingness (De Witte et al., 2018).

One of the most noteworthy findings of the present study is that even teachers with high attitude levels reported various challenges related to sustainability. This finding indicates that the factors influencing the sustainable use of assistive technologies are not limited to individual attitudes alone. The literature on sustainability in education emphasizes that the long-term continuation of effective practices is closely associated with institutional support, resource management, and system-level arrangements (Moore et al., 2017; Örücü, 2015). Similarly, the findings of the present study showed that, despite teachers’ positive attitudes toward assistive technologies, deficiencies in institutional support created barriers to their sustainable use. This finding may be considered one of the most important contributions of the present study to the existing literature. While the majority of studies in the field of assistive technologies have focused on the positive influence of teachers’ attitudes toward technology on technology use (Aslan and Kan, 2017; Demirok et al., 2019; Memet and Şentürk, 2021), the present study demonstrates that although positive attitudes are necessary for sustainable use, they are not sufficient on their own. In other words, teachers’ development of positive attitudes toward assistive technologies does not guarantee the long-term and continuous use of these technologies in educational settings. This finding suggests that the sustainable use of assistive technologies is shaped by the interaction of multiple factors, including institutional support, technical infrastructure, professional development opportunities, and the implementation context, in addition to individual attitudes.

This finding is also consistent with the Normalization Process Theory (May and Finch, 2009). According to this theory, the sustainability of new practices depends not only on individuals’ adoption of these practices but also on their integration into routine work processes, institutional support, and continuous evaluation. In the present study, the fact that even teachers with high attitude levels reported deficiencies in technical support and institutional infrastructure indicates that the sustainable use of assistive technologies cannot be explained solely by individual acceptance.

When the views of teachers with high and low attitude levels were compared, important differences emerged. Teachers with high attitude levels perceived the challenges they encountered as manageable problems, whereas teachers with low attitude levels described the same challenges as major barriers to the use of assistive technologies. This finding suggests that teachers’ attitudes may play a protective role in the sustainable use of technology. In other words, teachers with positive attitudes appear to make greater efforts to continue using assistive technologies and are more willing to develop solutions to the challenges they encounter. In contrast, negative or low attitude levels may lead to the gradual abandonment of technology over time (Howard, 2013).

Another important finding of the present study concerns the influence of in-service training on sustainability. During the interviews, many teachers reported that practice-oriented training on assistive technologies increased both the frequency of their technology use and their self-confidence. This finding is consistent with previous studies demonstrating the relationship between teacher competence and technology use (Ertmer and Ottenbreit-Leftwich, 2010; Instefjord and Munthe, 2017; Philipsen et al., 2019; Sertkaya, 2021; Tondeur et al., 2017; Uerz et al., 2018). Ensuring teachers’ access to assistive technologies alone is not sufficient for their effective and sustainable use. Rather, teachers also require continuous professional support on how to integrate these technologies into instructional processes.

Overall, the findings of the present study indicate that the sustainable use of assistive technologies is a multidimensional process. Although teachers’ attitudes constitute an important component of this process, sustainability is achieved through the combined influence of teacher competencies, institutional support mechanisms, technical infrastructure, maintenance and repair processes, and professional development opportunities. Therefore, both individual- and system-level factors should be considered together to ensure the long-term and effective use of assistive technologies in education.

This study makes an original contribution to the literature by explaining the sustainable use of assistive technologies not only from the perspective of technology acceptance but also by considering teachers’ experiences together with contextual factors. Furthermore, the use of an explanatory sequential mixed methods design made it possible to explain the underlying reasons for the quantitatively identified teacher attitudes through qualitative findings, thereby contributing to a more comprehensive understanding of the sustainable use of assistive technologies.

## 5 Recommendations

The findings of the present study indicate that the sustainable use of assistive technologies depends not only on teachers’ positive attitudes but also on institutional support, technical infrastructure, and opportunities for professional development. Accordingly, it is recommended that practice-oriented in-service training programs for teachers working with students with severe and multiple disabilities be expanded, technical support mechanisms in schools be strengthened, and assistive technologies be systematically integrated into school policies and administrative processes. In addition, establishing professional learning communities that support teachers’ exchange of experiences may contribute to the dissemination of good practices and promote the sustainable use of assistive technologies.

Future research should compare the experiences of teachers working with different disability groups and examine the relationships among teachers’ attitudes, technology use, self-efficacy, and technology acceptance within the framework of different theoretical models. Furthermore, it is recommended that multi-stakeholder studies involving school administrators, families, support personnel, and students be conducted, and that intervention programs designed to enhance the sustainable use of assistive technologies be evaluated through longitudinal, experimental, or mixed methods research.

## 6 Limitations

The findings of the present study should be interpreted in light of several limitations. First, the study was conducted exclusively with special education teachers working with students with severe and multiple disabilities in Türkiye; therefore, the findings cannot be directly generalized to different educational systems or cultural contexts. In addition, the quantitative data were collected based on teachers’ self-reports, and the actual frequency and sustainability of assistive technology use were not assessed through objective measures. Therefore, future studies may provide more comprehensive evidence by incorporating multiple data sources, such as classroom observations, usage records, and digital usage data.

The cross-sectional design of the study does not allow changes in teachers’ attitudes and the sustainable use of assistive technologies to be examined over time. Furthermore, the qualitative phase included only teachers’ perspectives, and participants were selected through purposive sampling based on the quantitative findings, consistent with the explanatory sequential mixed methods design. As a result, the perspectives of other stakeholders and the full diversity of teacher profiles may not have been represented. Although an inferential comparison was conducted based on AT-specific training status, the quantitative phase did not comprehensively examine differences in teachers’ attitudes across other demographic characteristics. Future studies may employ more extensive inferential and multivariate analyses to investigate the potential influence of demographic and professional variables on teachers’ attitudes toward assistive technologies. Future longitudinal, multi-stakeholder, and quantitatively driven studies employing different analytical models may contribute to a more comprehensive understanding of the individual and contextual processes influencing the sustainable use of assistive technologies.

## 7 Conclusion

This study revealed that special education teachers working with students with severe and multiple disabilities generally have high levels of positive attitudes toward assistive technologies. However, the qualitative findings demonstrated that although positive attitudes constitute an important factor supporting the sustainable use of assistive technologies, they are not sufficient on their own. Teachers reported that assistive technologies make significant contributions to the communication, participation, and independence of students with severe and multiple disabilities. At the same time, they identified the lack of technical support, maintenance and repair problems, insufficient in-service training, and limited institutional resources as major barriers to the sustainable use of these technologies. The findings indicate that the sustainable use of assistive technologies is a multidimensional process shaped by the interaction of individual, pedagogical, and institutional factors. In this context, sustainability appears to depend not only on introducing technological tools into educational settings but also on establishing systematic structures that support their continued use. This study not only identified teachers’ attitudes toward assistive technologies but also comparatively examined how teachers with different attitude levels experience the sustainable use of assistive technologies.

## Data Availability

The datasets generated and analyzed during this study are not publicly available because they contain confidential information obtained from human participants and are protected under the conditions of the ethical approval. Data may be made available from the corresponding author (htorunyeterge@adiyaman.edu.tr) upon reasonable request, provided that the request complies with the ethical approval and participant confidentiality requirements. Requests to access the datasets should be directed to htorunyeterge@adiyaman.edu.tr.
